# Evaluation of swabbing methods for culture and non-culture-based recovery of multidrug-resistant organisms from environmental surfaces

**DOI:** 10.1017/ice.2025.10214

**Published:** 2025-08

**Authors:** Ahmed Babiker, Julia Van Riel, Sarah Lohsen, Alex Page, Amanda Strudwick, Eli Wilber, Michael Woodworth, Sarah Satola

**Affiliations:** Division of Infectious Diseases, Department of Medicine, Emory University School of Medicine, Atlanta, GA, USA

## Abstract

**Objectives:**

Sponge-Sticks (SS) and ESwabs are frequently utilized for detection of multidrug-resistant organisms (MDROs) in the environment. Head-to-head comparisons of SS and ESwabs across recovery endpoints are limited.

**Design:**

We compared MDRO culture and non-culture-based recovery from (1) ESwabs, (2) cellulose-containing SS (CS), and (3) polyurethane-containing SS (PCS).

**Methods:**

Known quantities of each MDRO were pipetted on a stainless-steel surface and swabbed by each method. Samples were processed, cultured, and underwent colony counting. DNA was extracted from sample eluates, quantified, and underwent metagenomic next-generation sequencing (mNGS). MDROs underwent whole genome sequencing (WGS). MDRO recovery from paired patient perirectal and PCS-collected environmental samples from clinical studies was determined.

**Setting:**

Laboratory experiment, tertiary medical center, and long-term acute care facility.

**Results:**

Culture-based recovery varied across MDRO taxa, it was highest for vancomycin-resistant *Enterococcus* and lowest for carbapenem-resistant *Pseudomonas aeruginosa* (CRPA). Culture-based recovery was significantly higher for SS compared to ESwabs except for CRPA, where all methods performed poorly. Nucleic acid recovery varied across methods and MDRO taxa. Integrated WGS and mNGS analysis resulted in successful detection of antimicrobial resistance genes, construction of high-quality metagenome-assembled genomes, and detection of MDRO genomes in environmental metagenomes across methods. In paired patient and environmental samples, multidrug-resistant *Pseudomonas aeruginosa (*MDRP) environmental recovery was notably poor (0/123), despite detection of MDRP in patient samples (20/123).

**Conclusions:**

Our findings support the use of SS for the recovery of MDROs. Pitfalls of each method should be noted. Method selection should be driven by MDRO target and desired endpoint.

## Background

Contaminated surfaces within the healthcare environment play an important role in patient acquisition and transmission of multidrug-resistant organisms (MDROs).^1^ Environmental surface sampling for the recovery of pathogens is indicated in select settings such as during an outbreak investigation to identify routes of transmission that may require targeted interventions and/or for healthcare epidemiology research purposes.^2^

Sample/rinse methods are frequently utilized for environmental sampling due to their versatility and ease of use.^2,3^ ESwab and Sponge-Stick (SS) sampling methods are frequently utilized and recommended by public health agencies.^2^ Theoretical advantages and disadvantages exist for both methods.^3^ SS allow for a larger surface area of sampling and application of pressure and have in some settings been shown to have improved recovery over other methods.^3^ Despite this, similar recovery is potentially achievable with ESwabs.^4,5^ Moreover, ESwabs require less processing time, less specialized equipment and in theory are optimized for molecular workflows having previously been used for environmental microbiome analysis.^6^

Robust head-to-head comparisons of these methods across different recovery endpoints is limited. No evaluation of different SS compositions (polyurethane vs. cellulose) has been performed. Thus, we aimed to compare the effectiveness of three sampling methods; ESwabs, cellulose-containing SS (CS), and polyurethane-containing SS (PCS)—to recover MDROs from a contrived contaminated environmental surface.

## Methods

A controlled experiment of MDRO recovery with three collection methods was conducted: (1) ESwabs moistened with ESwab solution (Copan Diagnostics Inc. Murrieta, CA, USA), (2) CS (3M Company, Maplewood, MN, U.S.) and (3) PCS (Hygienia, Camarillo, CA. USA). Our study design was informed by a pilot study we conducted prior where cotton swabs moistened with phosphate buffer solution (PBS) was additionally evaluated. Given the poor performance of cotton swabs, it was not further evaluated.^7^

MDRO strains used as positive controls included clinical strains of extended spectrum ß-lactamase (ESBL) producing *Escherichia coli*, carbapenem-resistant *Acinetobacter baumanii* (CRAB) and vancomycin-resistant *Enterococcus* (VRE), a CDC antibiotic resistance (AR) bank carbapenem-resistant *Pseudomonas aeruginosa* (CRPA) isolate (AR0054) and an American Type Culture Collection (ATCC) methicillin-resistant *Staphylococcus aureus* (MRSA) control strain (ATCC 33591).

Dilutions containing 10^8^ CFU per milliliter (CFU/mL) were made for each MDRO strain. Input colony count was obtained for each strain by serially diluting and plating in 10 μL drops in quadruplicate. Colony counts were then averaged and used to calculate the estimated input CFU/mL for each round of testing. One mL of the bacterial solution was then dispensed over four stainless steel surfaces (8 in × 12 in) in 5 μL dots and allowed to dry for 1 h. Hand hygiene and appropriate PPE and gloves were donned prior to sampling. Testing of swabbing methods happened in parallel and were performed in triplicate by three swabbers resulting in three replicates for each method–organism-swabber combination. The sampling surface was disinfected with bleach and cleaned with 70% ethanol before and after each swabbing round.

SS sampling consisted of using one wide side of the sponge to wipe across the surface first in a horizontal motion, followed by sampling in a vertical motion using the other wide side, then using the narrow sides to sample in a diagonal motion, rotating the sponge halfway, and finally using the tip to wipe the perimeter of the swabbing area.^8^ ESwab sampling consisted of using the swab to wipe across the surface first in a horizontal motion, followed by a vertical motion, followed by a diagonal motion, rolling the back and forth to ensure that all sides of the swab made contact with the surface and that a maximal surface area was covered as previously described.^4^ ESwabs were only moistened once prior to sampling.

### Culture-based recovery

**Figure 1.**
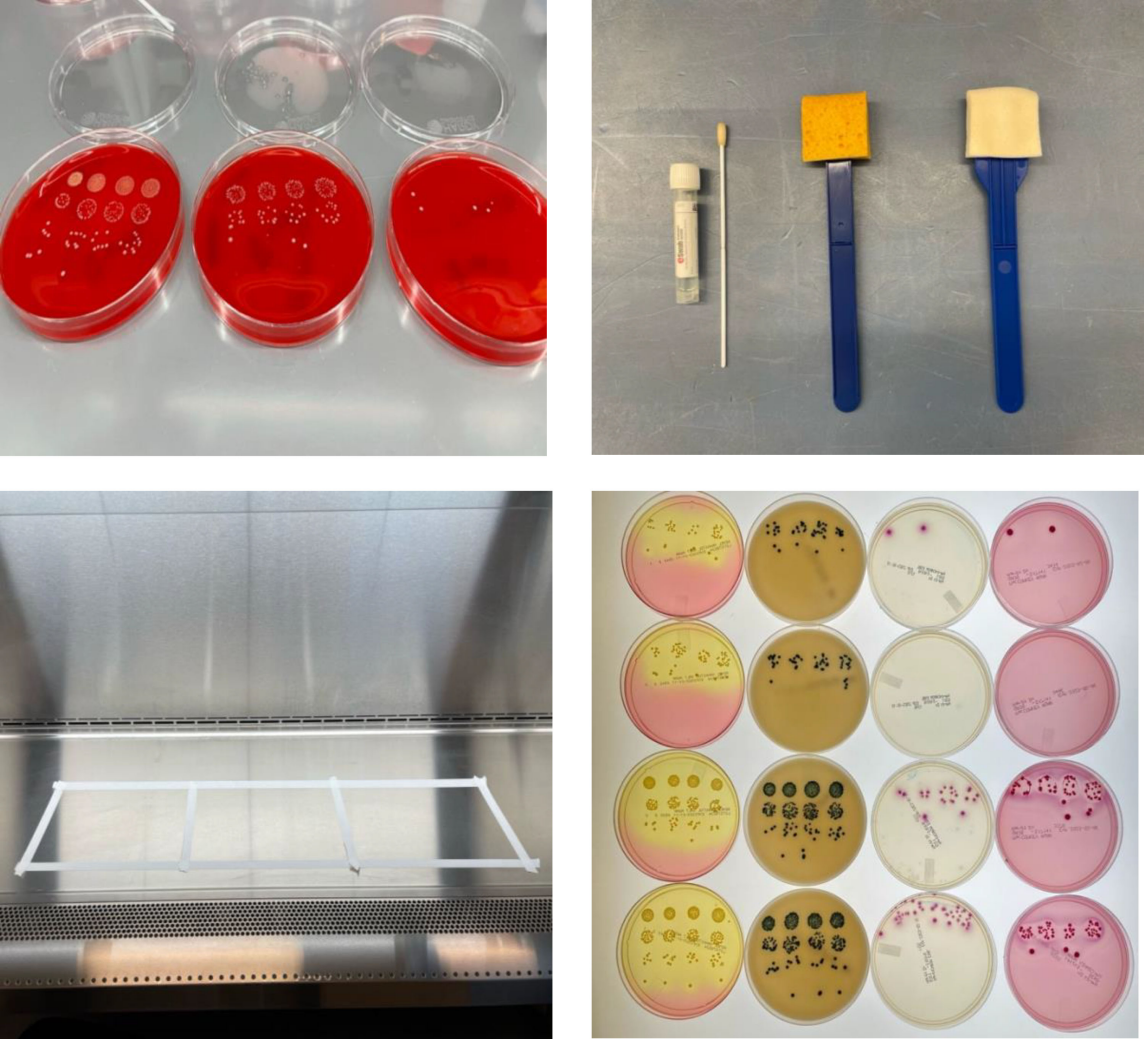
Photographs illustrating methods to assess the effectiveness of surface sampling for culture and metagenomic analysis. (A) example of quadruplicate bacterial dilution plating to determine starting inoculum and post-swabbing colony recovery; (B) Different sampling methods assessed (from left to right) Ewab, cellulose containing sponge-sticks, polyurethane containing sponge-sticks (C) 8 inch by 12 inch stainless steel surfaces which bacterial suspension was applied to (D) MDRO colonies on selective and differential chromogenic agars (from right to left) salt-mannitol MRSA selective agar, VRE selective agar, ESBL selective agar, MacConkey agar.

SS were expressed in PBS containing 0.02% Tween 80 using a stomacher, centrifuged, and resuspended in PBS; ESwabs were vortexed. 10 μL of fluid from each method was serially diluted and plated on selective and nonselective media in quadruplicate and incubated at 37 °C for 24 h. Viable colonies were then counted (Figure 1).

### Nucleic acid recovery

Residual PBS for SS and ESwab fluid for ESwabs was extracted using the Magmax Viral/Pathogen Kit on the Kingfisher Apex (ThermoFisher Scientific, Waltham, MA, USA). Extracted genomic material was quantified with Nanodrop (ThermoFisher Scientific, Waltham, MA, USA). Samples planned for mNGS analysis underwent DNA extractions using the ZymoBIOMICS™ DNA Miniprep Kit2 (Zymo Research, Irvine, CA, USA).

### Integrated genomic and metagenomic analysis

Pure isolates of MDRO strains underwent whole genome sequencing (WGS). Highest concentration samples for each sampling method and MDRO taxa combination along with negative controls were selected for metagenomic next-generation sequencing (mNGS). Illumina sequencing libraries were prepared using the M tagmentation-based and PCR-based Illumina DNA Prep kit (Illumina, San Diego, CA, USA) and custom IDT 10bp unique dual indices with a target insert size of 280 bp. Illumina sequencing was performed on an Illumina NovaSeq X Plus sequencer in one or more multiplexed shared-flow-cell runs, producing 2x151bp paired-end reads. Demultiplexing, quality control and adapter trimming was performed with bcl-convert (v4.2.4).

### Bioinformatic analysis

Genomes and metagenomes underwent analysis as previously described.^9^ Full bioinformatic methods are described in the supplemental materials. FASTQ files were deposited in the sequence read archive (BioProject ID PRJNA1224773).

### Studies of paired patient and sponge-stick collected environmental samples

We determined MDRO environmental recovery from four ongoing clinical studies of paired perirectal and environmental samples. These included (1) a case-control study of MDRO infected patients and unit-based negative controls,^10^ (2) a facility wide point prevalence sampling of long-term acute care facility (LTACH) patients,^11^ (3) an open-label trial of fecal microbiota transplantation (FMT) for the eradication of MDRO colonized LTACH patients (NCT05780801), (4) a phase 2, randomized controlled trial of FMT for the treatment of MDRO colonization (NCT05835206).

In all the studies sample collection and processing was standardized. Perirectal (PR) samples were collected using ESwabs and composite environmental samples were collected using PCS as per the protocol adopted from.^12^ Composite 1 included the TV remote, telephone, call button, and bed rails. Composite 2 included the room door handle, IV pole, and overbed table. Composite 3 included toileting surfaces if used by the patient. The sampled area did not exceed 350 mm^2^. In the abovementioned studies two and three, only composite 1 was collected. PCS samples were processed as above targeting CRAB, carbapenem-resistant Enterobacterales (CRE), ESBL, MDRP, and VRE. MRSA was not a target organism. PCS and ESwabs were processed as described above.

### Statistical analysis

Percentage recovery (as compared to starting inoculum) and nucleic acid recovery (ng/μL) of each sample were calculated. Percent recovery and nucleic acid recovery across methods and MDRO taxa were compared using the Kruskal-Wallis rank and Wilcoxon rank sum test as appropriate. Sensitivity and negative predictive value (NPV) of each environmental swabbing method’s ability to detect AR genes was calculated using epiR package (https://cran.r-project.org/web/packages/epiR/epiR.pdf) with the MDRO genome results as the gold standard. Analysis, visualization of relative abundance and coverage breadth and depth and figures were produced in R using the R Studio interface.^13^

## Results

### Culture-based recovery

Culture-based recovery measured as a percentage varied significantly by MDRO taxa and sampling methods (*p* < 0.001) (Table 1). Across all methods, Gram-positive MDROs VRE and MRSA were recovered at the highest median [Q25, Q75] proportion (VRE percent recovery: 9.71% [0.50, 16.17], MRSA percent recovery: 2.26% [0.53, 4.30]) while CRPA (percent recovery: 0.00042% [0.00007, 0.00223]) was recovered at the lowest proportion.

**Table 1.** Percent recovery and nucleic acid yield for sampling methods

MDRO species	Sampling method					
	ESwab		Cellulose-containing Sponge-Stick		Polyurethane-containing Sponge-Stick	
	Median [q25, q75] Percent recovery (%)	Nucleic acid Concentration (ng/µl), Median [q25, q75]	Median [q25, q75] Percent recovery (%)	Nucleic acid Concentration (ng/µl), Median [q25, q75]	Median [q25, q75] Percent recovery (%)	Nucleic acid Concentration (ng/µl), Median [q25, q75]
CRAB	0.03 [0.01, 0.07]	3.32 [2.71, 4.68]	0.10 [0.08, 1.50]	12.13 [10.20, 15.57]	0.40 [0.21, 2.88]	4.96 [3.11, 5.30]
CRPA	0.0001 [0.00006, 0.0004]	3.73 [3.36, 4.57]	0.0002 [0.00005, 0.00360]	10.31 [7.01, 15.51]	0.0008 [0.0005, 0.0017]	3.21 [2.25, 3.37]
ESBL	0.16 [0.02, 0.20]	5.90 [3.09, 6.43]	1.80 [0.15, 3.43]	9.36 [4.59, 12.84]	1.93 [0.55, 6.60]	2.35 [2.05, 4.24]
MRSA	0.16 [0.04, 0.31]	4.97 [2.34, 7.31]	3.76 [3.42, 4.41]	23.18 [15.56, 26.75]	2.34 [2.20, 7.79]	12.81 [9.16, 14.70]
VRE	0.19 [0.09, 0.46]	4.72 [4.35, 4.74]	10.83 [9.50,11.96]	17.77 [14.63, 20.63]	17.50 [16.42, 24.59]	6.82 [5.88, 7.90]

Abbreviations: CFU: colony forming units, CRAB: carbapenem-resistant *Acinetobacter baumannii* complex, CRPA: carbapenem-resistant *Pseudomonas aeruginosa*, ESBL: extended spectrum beta-lactamase producing Enterobacterales, MDRO: multidrug-resistant organism, MRSA: methicillin-resistant *Staphylococcus aureus*, VRE: vancomycin-resistant enterococcus.

**Figure 2.**
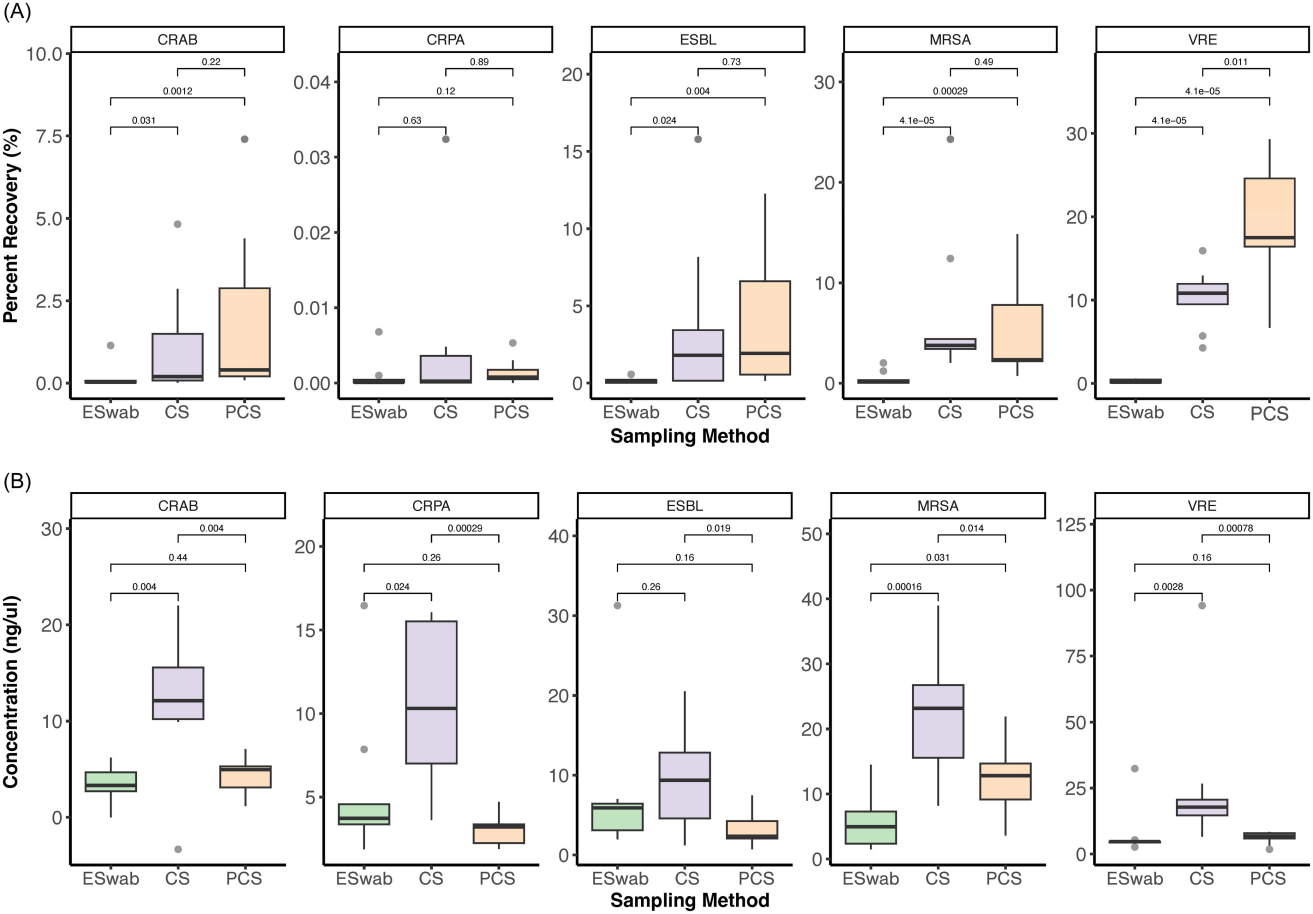
Recovery of percent of starting inoculum by culture (A), and nucleic acid concentration after extraction (B) for each MDRO category and sampling method. Abbreviations: CRAB: carbapenem-resistant *Acinetobacter baumannii*, CRPA: carbapenem-resistant *Pseudomonas aeruginosa*, CS: cellulose-containing Sponge-Stick, ESBL: extended spectrum beta-lactamase producing Enterobacterales, MRSA: Methicillin-resistant *Staphylococcus aureus* PCS: polyurethane-containing Sponge-Stick, VRE: vancomycin-resistant *Enterococcus*.

Across all MDRO taxa, culture-based recovery was significantly higher among SS (CS and PCS) compared to ESwabs except for CRPA (percent recovery: ESwab: 0.0001% [0.00006, 0.0004] *vs.* CS: 0.0002% [0.000055, 0.0036] *vs.* PCS: 0.0008% [0.0005, 0.0017], *p* = 0.42), where all three methods performed poorly (Table 1, Figure 2, Supplementary Figure 1A). Culture-based recovery of all MDRO taxa was similar across the two SS methods, except for VRE where PCS outperformed CS (percent recovery: CS: 10.83% [9.50, 11.96] *vs.* PCS: 17.50% [16.42, 24.59], *p* = 0.01) (Table 1, Figure 2).

### Nucleic acid recovery

Similar to culture-based recovery, nucleic acid yield significantly varied across MDRO taxa (*p* < 0.001) and swabbing method (*p* < 0.001) (Figure 2B, Table 1). Nucleic acid yield was highest for gram-positive MDROs (MRSA: 12.81 [5.50, 18.16] ng/μL, VRE: 6.82 [4.72, 15.95]) and lowest for the Gram-negative MDROs (median [IQR] nucleic acid yield: CRAB: 5.06 [3.13, 10.06] ng/μL, CRPA: 3.95 [3.28, 8.18] ng/μL, ESBL: 4.68 [2.26, 7.29] ng/μL) regardless of method.

Across most MDRO taxa, nucleic acid yield was significantly higher with CS compared to both ESwab and PCS, except for ESBL where it was similar to ESwab (ESwab: 5.89 [3.09, 6.43] ng/μL *vs.* CS: 9.36 [4.58, 12.84] ng/μL, *p* = 0.26; Figure 2B, Supplementary Figure 1B, Table 1).

### Integrated genomic and metagenomic recovery and analysis

Due to low culture- and non-cultured-based recovery of CRAB and CRPA across methods these samples did not undergo mNGS. ESBL, MRSA and VRE ESwabs, CS and PCS samples with the highest nucleic acid concentration and ESwab, CS and PCS negative controls underwent mNGS.

**Figure 3.**
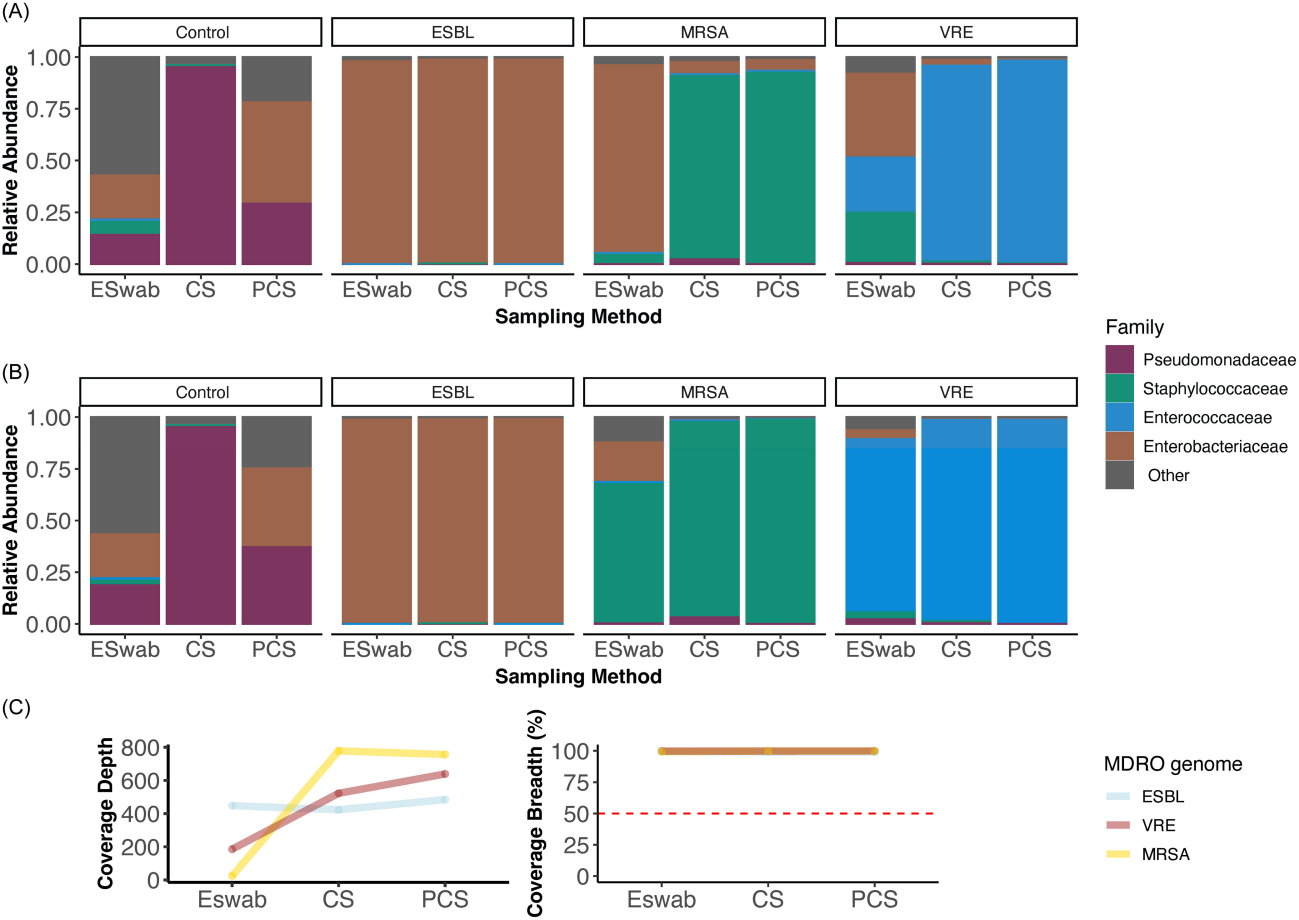
Family-level taxonomic relative abundance of metagenomes from each MDRO category positive control and sponge combination pre-(A) and post- (B) bioinformatic decontamination. Decontaminated metagenomes all had 0.6 proportional abundance of positive control MDRO genomes but sponge sticks were closer to 1.0 relative abundance, likely due to higher biomass and DNA yields. (C) MDRO genome coverage depth and breadth within each sampling method metagenome sequenced with equal target depth, demonstrating consistent 100% coverage breadth across methods but lower depth with ESwabs. Abbreviations: CS: cellulose-containing Sponge-Stick, ESBL: extended spectrum beta-lactamase producing Enterobacterales, MRSA: Methicillin-resistant *Staphylococcus aureus*, PCS: polyurethane-containing Sponge-Stick, VRE: vancomycin-resistant Enterococcus.

Metagenomic sequencing resulted in the generation of higher read count from the CS negative control compared to PCS and Eswab negative controls. (Supplementary table 1). Upon review of the relative abundance plots, contamination of the environmental metagenomes with off target species was detected (Figure 3A). This was further confirmed by the construction of off target metagenome-assembeled genomes (MAGs). Metagenomes underwent bioinformatic decontamination and relative abundance was recalculated and visualized (Figure 3B). ESwab metagenomes were most susceptible to contamination and most resistant to decontamination as evident on relative abundance plots. Decontaminated metagenomes all had 60% proportional abundance of positive control MDRO genomes but SS were closer to 100% relative abundance, likely due to higher biomass and DNA yields (Figure 3B). The CS negative control had close to 100% proportional abundance of *Pseudomonas spp* reads.

### AR gene detection

The major AR genotype and phenotype-defining genes detected in the MDRO genomes included a _bla_CTX-M-15 for the ESBL isolate, a blaZ and mecA gene for the MRSA isolate, and a vanA operon for the VRE isolate. These AR genes were detected in all metagenomes regardless of swabbing method. In total, there were 11 AR genes detected in the ESBL genome, 13 AR genes detected in the MRSA genome and 14 AR genes detected in the VRE genome. Sensitivity (range: 1.00 [0.77, 1.00] – 0.92 [0.64, 1.00] and NPV (range: 0.96 (0.82, 1.00] – 1.00 [0.89, 1.00) for AR gene detection was high across all swabbing methods (Table 2). No AR genes were detected in the negative control metagenomes.

**Table 2.** AR detection and metagenome assembled genomes quality metrics. MAGs were binned from assembled contigs with taxonomic classification for each contrived experiment metagenome. In each method and MDRO combination, a MAG was recovered with AR genes that corresponded to the known positive control isolate genome. These data demonstrate feasibility of culture-independent MDRO MAG recovery from environmental surfaces with metagenomic sequencing when DNA yields are adequate

	Swabbing Method and MDRO								
	ESwab			CS			PCS		
	ESBL	MRSA	VRE	ESBL	MRSA	VRE	ESBL	MRSA	VRE
AR gene detection sensitivity (95% CI)	1.00 (0.72, 1.00)	0.92 (0.64, 1.00)	1.00 (0.77, 1.00)	1.00 (0.72, 1.00)	1.00 (0.75, 1.00)	1.00 (0.77, 1.00	1.00 (0.72, 1.00)	1.00 (0.75, 1.00)	1.00 (0.77, 1.00)
AR Gene detection NPV (95% CI)	1.00 (0.89, 1.00)	0.96 (0.82, 1.00)	1.00 (0.74, 1.00)	1.00 (0.89, 1.00)	1.00 (0.87, 1.00)	1.00 (0.87, 1.00)	1.00 (0.89, 1.00)	1.00 (0.88, 1.00)	1.00 (0.88, 1.00)
MAG quality score	100	68	98	86	60	97	100	100	57
MAG quality category	High	Medium	High	High	High	High	High	High	Low
MAG size (bp)	4798235	1830370	2241406	5057975	2890163	2420310	4783087	2882677	557748
MAG completeness, %	99.99	58.83	98.1	100	100	99.98	100	100	27.87
MAG contamination, %	0.02	1.47	0.66	0.04	0.73	0.68	0.04	0.71	0
MAG contigs, N	74	228	96	86	60	97	79	43	39
MAG N50, bp	105231	9301	33236	105230	76026	37326	105230	145139	19229
Species	*Escherichia coli*	*Staphylococcus aureus*	*Enterococcus faecium*	*Escherichia coli*	*Staphylococcus aureus*	*Enterococcus faecium*	*Escherichia coli*	*Staphylococcus aureus*	*Enterococcus faecium*

Abbreviations: AR: antimicrobial resistance, BP: base pairs CI: confidence interval, CS: cellulose sponge-sticks: ESBL: extended spectrum beta-lactamase producing Enterobacterales, MDRO: multidrug-resistant organism, MRSA: methicillin-resistant *Staphylococcus aureus*, MAG: metagenome assembled genome NPV: negative predictive value, PCS: polyurethane sponge-sticks VRE: vancomycin-resistant enterococcus.

### MAG construction and genome tracking

To simulate culture agonistic mNGS recovery of MDROs from the environment, we *de novo* assembled MAGS from the environmental metagenomes. Across all swabbing methods, high-quality MAGs representing the target MDROs (ESBL, MRSA, and VRE) were constructed for all but two samples. These included a medium-quality MRSA ESwab MAG (bin score: 0.68, completeness: 58.83%) and low-quality PCS VRE MAG (bin score: 0.54, completeness: 8.52%). Improved MAG completeness was seen after decontamination and reconstruction of the PCS VRE MAG (bin score: 0.56, completeness: 27.8%) (Table 2). Attempts to construct MAGs from the negative control sample failed. MDRO isolate genomes were detected with 100% coverage breadth within environmental metagenomes across all methods but at a lower depth within the ESwab metagenomes (Figure 3C).

### MDRO recovery from paired patient and PCS collected environmental samples

Overall, 73 patients underwent paired perirectal sampling and environmental swabbing resulting in 123 paired environmental and perirectal samples (Table 3, Supplementary Figure 2). Among the 123 collected patient samples, 74.8% (92/123) had at least one target MDRO detected. Among those 33.7% (31/92) had the same MDRO (by phenotype) detected in the environment (Table 3).

**Table 3.** Detection of multidrug-resistant organisms in paired patient perirectal samples and polyurethane sponge-sticks collected environmental samples

	MDRO Category				
	CRAB	CRE	ESBL	MDRP	VRE
Detected in patient*	0% (0/123)	11.4% (14/123)	38.2% (47/123)	16.3% (20/123)	36.6% (45/123)
Detected in ENV*	0% (0/123)	6.5% (8/123)	5.7% (7/123)	0% (0/123)	26.0% (32/123)
Detected in both*	0% (0/123)	4.1% (5/123)	4.9% (6/123)	0% (0/123)	21.1% (26/123)
Detected in patient only*	0% (0/123)	7.3% (9/123)	33.3% (41/123)	16.3% (20/123)	15.4% (19/123)
Detected in ENV only*	0% (0/123)	2.4% (3/123)	0.8% (1/123)	0% (0/123)	4.9% (6/123)
Not detected*	0% (0/123)	86.2% (106/123)	61.0% (75/123)	83.7% (103/123)	58.5% (72/123)

* Categories are not mutually exclusive.

Abbreviations: CRAB: carbapenem-resistant *Acinetobacter baumannii* complex, CRE: carbapenem-resistant Enterobacterales, ENV: Environment, ESBL: extended spectrum beta-lactamase producing Enterobacterales, MDRO: multidrug-resistant organism, MDRP: multidrug-resistant *Pseudomonas aeruginosa*, MRSA: methicillin-resistant *Staphylococcus aureus*, VRE: vancomycin-resistant enterococcus.

Across studies, 38.4% (53/123), 36.6% (45/123), 16.3% (20/123), and 11.4% (14/123) patients had ESBL, VRE, MDRP, CRE detected respectively in their perirectal samples. No patients had CRAB detected (0/123). Among patients that had an MDRO detected, 57.8% (26/45), 35.7% (5/14), 11.3% (6/53), of patients with VRE, CRE and ESBL detected in their perirectal sample had the same phenotypic MDRO detected in the environment. Notably, no MDRP was detected in any of the environmental samples, including the environmental samples of the 20 patients that had MDRP detected in their perirectal samples.

## Discussion

We compared the culture and non-culture-based effectiveness of ESwabs, CS, and PCS to recover MDRO taxa from a contrived contaminated environmental surface. We found SS resulted in more efficient culture-based recovery than ESwabs. However, culture-based recovery was strongly dependent on MDRO taxa. Recovery of non-fermenter Gram-negative MDROs, in particular *Pseudomonas aeruginosa,* was poor across methods. This was reflected in our real-world data of paired PCS-collected environmental and patient samples where MDRP was detected in patients PR samples but was not detected in any of the environmental samples. This contrasted with other MDROs which were frequently recovered from proximal healthcare environmental surfaces when detected in patient samples. In integrated WGS and mNGS analysis of contrived samples all methods resulted in sensitive detection of major AR genes, *de-novo* assembly of MAGs and detection of the MDRO genomes within the metagenomes.

Our study is one of the first to compare SS of different compositions and ESwabs across endpoints. We found SS outperformed ESwab in terms of culture-based recovery. We hypothesize this may be due to larger surface area that SS can sample as they have a significantly larger functional surfaces for sampling. Prior studies have shown that culture-based recovery decreases with an increase in sampled surface area.^14^ Moreover, SS have a sturdy handle, which facilitates even application of pressure when compared to ESwabs (Figure 1).^2,3^ Importantly, SS composition did not have a major impact on culture-based recovery. Others have demonstrated similar recovery rates of ESwabs and SS in similar lab-based simulations and clinical studies.^4,15,16^ This may have been driven by the smaller area sampled in prior studies, as similar recovery is achievable when sampling a limited area.^14^ Hence, when sampling large surface areas, the SS is better suited. While the ESwab is a reasonable option when sampling a more restricted area (or when breaking down larger areas into smaller areas to allow for granular environmental contamination mapping). Furthermore, ESwabs have the advantage of getting into crevices, not requiring a stomacher and being more readily available in healthcare settings. All of these factors should be weighed when selecting a sampling method of choice.

We saw significant variation in both quantitative and qualitative culture-based recovery across MDRO taxa. This is likely due to organism-specific biological properties that influence environmental adherence and persistence.^17^ We noticed a significant lack of MDRP recovered from the environmental samples collected in rooms of MDRP-colonized patients. This was in contrast to other MDROs which were frequently detected across clinical contexts and methods. While MDRP is a known inhabitant of the healthcare environment^6,18^ implicated in healthcare-associated transmission,^19,20^ it likely has a more specific environmental niche when compared to Gram-positive and Enterobacterales MDROs.^6^ MDRP is frequently recovered from sink traps, ventilator equipment, and sites distant from the patient and in proximity to wastewater sites.^6,18–20^ Hence, one potential reason for our lack of MDRP recovery is the focus on proximal patient sampling scheme in our study, which did not include any sinks or ventilators.^6^ This finding highlights the need to consider target organisms when designing sampling strategies.

With the increasing use of mNGS in epidemiological investigations and AR surveillance validation of sampling methods for non-culture-based recovery are needed.^21^ With mNGS, one has the advantage of a broad approach that lends itself to the recovery of novel pathogens and/or pathogens not subject to routine surveillance.^22,23^ Nucleic acid yield was higher with SS compared to ESwabs, driven by the performance of CS where sequencing of the negative control resulted in generation of more reads and detection of Pseudomonas on taxonomic analysis. However, we were able to accurately detect high-consequence AR genes, detect target species of interest and construct high-quality MAGs (a proxy for cultured genomes) and track our genomes within the metagenomes across all methods. Moreover, our experience with contamination and our ability to decontaminate our sequence data using bioinformatic approaches highlights the feasibility of quieting noise that can be expected in real-world environmental samples or dealing with similar issues of contamination with this nonspecific modality. ESwab metagenomes appeared to possibly be more susceptible to contamination and more resistant to decontamination. However, this finding will need to be further examined and validated in future studies.

Our study has some limitations we must acknowledge. Firstly, we did not include *Clostridioides difficile* and RODAC plates in our evaluation despite their respective roles in environmental contamination and sampling.^1,15^ Second, we utilized nanodrop and not Qubit for DNA quantification due to availability in our lab. Third, we did not evaluate other non-stainless-steel surfaces, the composition of which may impact yield.^17^ Fourth, like others we did not account for important variables such as timing of cleaning, quality of cleaning, patient duration in the room.^12,24^ Finally, our contrived environmental metagenomes may not fully reflect the true nature of sampling a non-sterile surface. However, our ability to track our genomes with 100% breadth was reassuring that this may be a feasible approach with a more complex sample. Further studies are needed to assess the robustness of our findings in real-world scenarios across a range of target organisms and sampling methods.

Our study adds to the growing body of literature attempting to guide infection prevention teams and researchers in selecting the appropriate recovery method which fits their intended use case. Recovery endpoint, target organisms, and surfacing sampling size should all be taken into consideration when selecting an environmental sampling method.

## Supporting information

Babiker et al. supplementary material Babiker et al. supplementary material

## Supplementary material

To view supplementary material for this article, please visit https://doi.org/10.1017/ice.2025.10214
