# Supplementary material for: Evaluation of swabbing methods for culture and non-culture-based recovery of multidrug-resistant organisms from environmental surfaces
Source: Infect Control Hosp Epidemiol. 2025 Jul 14;46(8):837–44. doi: 10.1017/ice.2025.10214 (PMC12422522; doi:10.1017/ice.2025.10214)
Supplement: Babiker et al. supplementary material [file S0899823X25102146sup001.docx]

**Supplemental Material**

*Bioinformatic Analysis*

Genomes and metagenomes underwent analysis as previously described^1^. Briefly, FASTQ files underwent processing using Trimmomatic v0.39^2^. Taxonomic classification was performed with kraken2 v1.1.1^3^. Genomes were assembled with SPAdes v3.15.5 and metagenomes were assembled with the —meta parameter. Assembly was evaluated using Quast and MetaQUAST v5.2.0^4^. Contigs were gene prediction was performed using Prodigal V.2.6.3^5^ and annotated with Prokka v1.14.6^6^. AR gene detection was performed using AMRfinderPlus V.2.6.3 filtering for >90% identity, >90% AR gene match length. Metagenomes underwent short read taxonomic classification with kraken2 v1.1.1^3^, followed by Bayesian reestimation of abundance at the species level using bracken v2.6.1^7^. The average genome size and number of genome equivalents for each metagenome was estimated using MicrobeCensus^8^. Metagenome assembled genomes (MAGs) were constructed by binning contigs with MaxBin2 v2.2.7^9^ and MetaBAT2 v2.12.1^10^ and dereplicated, aggregated and scored using DAS Tool v1.1.7^11^. MAG completeness and contamination were assessed with CheckM2 v1.0.2^12^ and taxonomy assignment was performed with GTDB-Tk^13^. MAGs were classified as high quality (completion > 90%, contamination <5%), medium quality (completion 50-89%, contamination <10%), and low quality (completion <50%, contamination <10%)^14^ . Metagenome decontamination was performed by concatenating the contaminant MAGs and depleting the MAG reads using BBDuk version 37.62 ^15^. We calculated MDRO genome breadth (percentage of genome covered by at least one read) and coverage depth from mapped metagenome reads to assess whether specific MDRO strains were detectable in the metagenomes. Genome coverage breadth of 50% was used as a strain detection cutoff^16^. FASTQ files were deposited in the sequence read archive (BioProject ID PRJNA1224773).

**
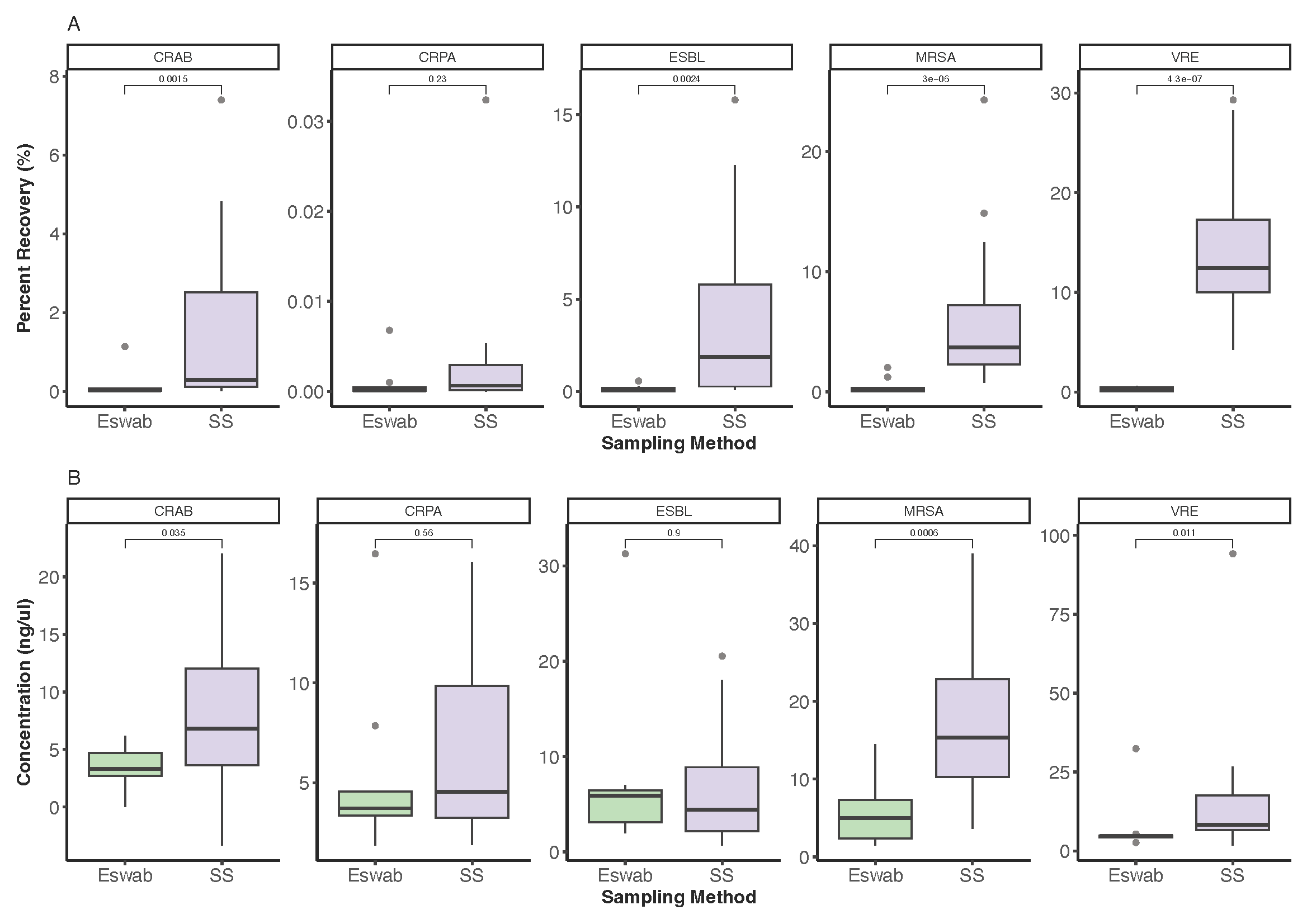
**

**Supplementary Figure 1.** Culture-based percent recovery (A) and nucleic acid extraction yield (B) for each MDRO category for sponge-stick vs. Eswabs. Note: y-axis values are distinct for each facet to illustrate dynamic range for each combination of swab and MDRO category.

Abbreviations: CRAB: carbapenem-resistant *Acinetobacter baumannii* complex, CRPA: carbapenem-resistant *Pseudomonas aeruginosa*, MDRO: multidrug-resistant organism, ESBL: extended spectrum beta-lactamase producing Enterobacterales, MRSA: methicillin-resistant *Staphylococcus aureus*, SS: sponge-stick, VRE: vancomycin-resistant enterococcus

| **Sample Name** | **Input sample concentration (ng/µl)** | **Total Read Pairs** | **Total Reads**  **(R1 + R2)** | **Total bp > Q30** | **bp > Q30 (%)** | **MDRO Category** | **Swab** |
| --- | --- | --- | --- | --- | --- | --- | --- |
| ESBL CS | 31.26 | 9653412 | 19306824 | 2690000000 | 94.872 | ESBL | CS |
| ESBL Eswab | 20.53 | 10719411 | 21438822 | 2990903204 | 95.918 | ESBL | Eswab |
| ESBL PCS | 7.473 | 12257281 | 24514562 | 3340000000 | 95.801 | ESBL | PCS |
| MRSA CS | 14.49 | 11680047 | 23360094 | 3205987228 | 96.257 | MRSA | CS |
| MRSA Eswab | 38.97 | 5979657 | 11959314 | 1613026574 | 94.328 | MRSA | Eswab |
| MRSA PCS | 21.92 | 10391260 | 20782520 | 2902020786 | 96.251 | MRSA | PCS |
| VRE CS | 32.38 | 6914827 | 13829654 | 1940000000 | 95.293 | VRE | CS |
| VRE Eswab | 94.15 | 9407854 | 18815708 | 2617295331 | 95.987 | VRE | Eswab |
| VRE PCS | 8.378 | 8959139 | 17918278 | 2490000000 | 95.529 | VRE | PCS |
| PCS negative control | ND | 21075 | 42150 | 4851160 | 93.551 | N/A | PCS |
| Eswab negative control | ND | 5655 | 11310 | 1385168 | 89.253 | N/A | Eswab |
| CS negative control | ND | 1929 | 3858 | 453198 | 79.744 | N/A | CS |

**Supplementary Table 1.** Raw sequencing statistics for environmental metagenomes. FASTQ files were deposited in the sequence read archive (BioProject ID PRJNA1224773).

Abbreviations: BP: base pairs CS: cellulose sponge-sticks: ESBL: extended spectrum beta-lactamase producing Enterobacterales, MDRO: multidrug-resistant organism, MRSA: methicillin-resistant *Staphylococcus aureus*, PCS: polyurethane sponge-sticks VRE: vancomycin-resistant enterococcus

**References**

**1.** Woodworth MH, Conrad RE, Haldopoulos M, et al. Fecal microbiota transplantation promotes reduction of antimicrobial resistance by strain replacement. *Sci Transl Med* 2023;15:eabo2750.

**2.** Bolger AM, Lohse M, Usadel B. Trimmomatic: a flexible trimmer for Illumina sequence data. *Bioinformatics* 2014;30:2114-2120.

**3.** Wood DE, Lu J, Langmead B. Improved metagenomic analysis with Kraken 2. *Genome Biology* 2019;20:257.

**4.** Mikheenko A, Saveliev V, Gurevich A. MetaQUAST: evaluation of metagenome assemblies. *Bioinformatics* 2015;32:1088-1090.

**5.** Hyatt D, Chen GL, Locascio PF, Land ML, Larimer FW, Hauser LJ. Prodigal: prokaryotic gene recognition and translation initiation site identification. *BMC Bioinformatics* 2010;11:119.

**6.** Seemann T. Prokka: rapid prokaryotic genome annotation. *Bioinformatics* 2014;30:2068-2069.

**7.** Lu J, Breitwieser FP, Thielen P, Salzberg SL. Bracken: estimating species abundance in metagenomics data. *PeerJ Computer Science* 2017;3:e104.

**8.** Nayfach S, Pollard KS. Average genome size estimation improves comparative metagenomics and sheds light on the functional ecology of the human microbiome. *Genome Biology* 2015;16:51.

**9.** Wu Y-W, Simmons BA, Singer SW. MaxBin 2.0: an automated binning algorithm to recover genomes from multiple metagenomic datasets. *Bioinformatics* 2015;32:605-607.

**10.** Kang DD, Li F, Kirton E, et al. MetaBAT 2: an adaptive binning algorithm for robust and efficient genome reconstruction from metagenome assemblies. *PeerJ* 2019;7:e7359.

**11.** Sieber CMK, Probst AJ, Sharrar A, et al. Recovery of genomes from metagenomes via a dereplication, aggregation and scoring strategy. *Nat Microbiol* 2018;3:836-843.

**12.** Chklovski A, Parks DH, Woodcroft BJ, Tyson GW. CheckM2: a rapid, scalable and accurate tool for assessing microbial genome quality using machine learning. *Nature Methods* 2023;20:1203-1212.

**13.** Chaumeil P-A, Mussig AJ, Hugenholtz P, Parks DH. GTDB-Tk v2: memory friendly classification with the genome taxonomy database. *Bioinformatics* 2022;38:5315-5316.

**14.** Bowers RM, Kyrpides NC, Stepanauskas R, et al. Minimum information about a single amplified genome (MISAG) and a metagenome-assembled genome (MIMAG) of bacteria and archaea. *Nature Biotechnology* 2017;35:725-731.

**15.** Bushnell B, Rood J, Singer E. BBMerge - Accurate paired shotgun read merging via overlap. *PLoS One* 2017;12:e0185056.

**16.** Olm MR, Crits-Christoph A, Bouma-Gregson K, Firek BA, Morowitz MJ, Banfield JF. inStrain profiles population microdiversity from metagenomic data and sensitively detects shared microbial strains. *Nature Biotechnology* 2021;39:727-736.
